# Resistance Toward Chlorhexidine in Oral Bacteria – Is There Cause for Concern?

**DOI:** 10.3389/fmicb.2019.00587

**Published:** 2019-03-22

**Authors:** Fabian Cieplik, Nicholas S. Jakubovics, Wolfgang Buchalla, Tim Maisch, Elmar Hellwig, Ali Al-Ahmad

**Affiliations:** ^1^Department of Conservative Dentistry and Periodontology, University Medical Center Regensburg, Regensburg, Germany; ^2^Centre for Oral Health Research, School of Dental Sciences, Newcastle University, Newcastle upon Tyne, United Kingdom; ^3^Department of Dermatology, University Medical Center Regensburg, Regensburg, Germany; ^4^Department of Operative Dentistry and Periodontology, Center for Dental Medicine, Faculty of Medicine, University of Freiburg, Freiburg im Breisgau, Germany

**Keywords:** antiseptic, chlorhexidine, CHX, resistance, adaptation, tolerance, persistence, efflux pump

## Abstract

The threat of antibiotic resistance has attracted strong interest during the last two decades, thus stimulating stewardship programs and research on alternative antimicrobial therapies. Conversely, much less attention has been given to the directly related problem of resistance toward antiseptics and biocides. While bacterial resistances toward triclosan or quaternary ammonium compounds have been considered in this context, the bis-biguanide chlorhexidine (CHX) has been put into focus only very recently when its use was associated with emergence of stable resistance to the last-resort antibiotic colistin. The antimicrobial effect of CHX is based on damaging the bacterial cytoplasmic membrane and subsequent leakage of cytoplasmic material. Consequently, mechanisms conferring resistance toward CHX include multidrug efflux pumps and cell membrane changes. For instance, in staphylococci it has been shown that plasmid-borne *qac* (“quaternary ammonium compound”) genes encode Qac efflux proteins that recognize cationic antiseptics as substrates. In *Pseudomonas stutzeri*, changes in the outer membrane protein and lipopolysaccharide profiles have been implicated in CHX resistance. However, little is known about the risk of resistance toward CHX in oral bacteria and potential mechanisms conferring this resistance or even cross-resistances toward antibiotics. Interestingly, there is also little awareness about the risk of CHX resistance in the dental community even though CHX has been widely used in dental practice as the gold-standard antiseptic for more than 40 years and is also included in a wide range of oral care consumer products. This review provides an overview of general resistance mechanisms toward CHX and the evidence for CHX resistance in oral bacteria. Furthermore, this work aims to raise awareness among the dental community about the risk of resistance toward CHX and accompanying cross-resistance to antibiotics. We propose new research directions related to the effects of CHX on bacteria in oral biofilms.

## Introduction

The 2016 Review on Antimicrobial Resistance has predicted an alarming scenario that the number of annual deaths attributable to antimicrobial resistance will increase globally from the current 700,000 to 10 million in the year 2050 (O’Neill, 2016). In this light, resistance toward antibiotics has attracted strong interest in the scientific as well as the medical community during the last few decades, and provided a major stimulus for establishing stewardship programs (Allerberger et al., 2009) and in the search for alternative antimicrobial measures such as antimicrobial peptides, natural compounds, cold atmospheric plasma, or light-based approaches (Karygianni et al., 2015; Czaplewski et al., 2016; Wainwright et al., 2017; Cieplik et al., 2018a). Furthermore, the oral cavity has recently been highlighted as a potential reservoir of antibiotic resistance genes that can be transferred *via* horizontal gene transfer among the bacteria present in oral biofilms (Roberts and Mullany, 2010; Al-Ahmad et al., 2014; Jiang et al., 2018).

Conversely, much less attention has been paid to the directly related problem of resistance toward the different classes of antiseptics and biocides (Forman et al., 2016; Kampf, 2016; Venter et al., 2017). This is somewhat surprising as bacterial resistance toward commonly used antiseptics such as benzalkonium chloride (BAC; a quaternary ammonium compound, QAC), triclosan (TCS; a polychloro phenoxy phenol) and chlorhexidine (CHX), as well as induction of cross-resistances between these agents and a range of clinically important antibiotics, have been known for many years (Yamamoto et al., 1988; McDonnell and Russell, 1999; Stickler, 2002; Russell, 2004; Yazdankhah et al., 2006).

While TCS has already been banned from its use in household wash products by the FDA in 2016 due to its risk of triggering cross-resistances (McNamara and Levy, 2016) and resistance toward QACs like BAC has also been highlighted in numerous reports (e.g., Buffet-Bataillon et al., 2012; Jaglic and Cervinkova, 2012), CHX has come into the spotlight only recently (Kampf, 2016; Venter et al., 2017; Wand et al., 2017). For example, it was reported that exposure to CHX may be associated with emergence of stable resistance to the last-resort antibiotic colistin, which may be associated with mutations in the two-component regulator *phoPQ* (Wand et al., 2017). The lack of focus on CHX resistance is somewhat surprising since it was found as early as 1980 that the extensive and liberal use of CHX in the management of patients with long-term bladder catheters resulted in urinary tract infections from CHX-resistant Gram-negative bacteria (i.e., strains of *Proteus mirabilis*, *Providencia stuartii*, and *Pseudomonas aeruginosa*), which were also found to be multidrug-resistant (Stickler and Thomas, 1980; Stickler, 2002). More recently, a high prevalence of reduced susceptibility toward CHX (69%) was found in microorganisms that caused central line-associated bloodstream infections in a large academic medical center with significantly higher proportion in patients who received daily CHX bathing (86% vs. 64%; *p* = 0.028) (Suwantarat et al., 2014).

Interestingly, the use of CHX in the field of dentistry for oral biofilm control has not been considered so far in this context as a possible source for the development of resistance against CHX itself or cross-resistances against other antiseptics or antibiotics (Sreenivasan and Gaffar, 2002). This is in spite of the widespread use of CHX by dental clinicians as gold-standard antiseptic (Jones, 1997), e.g., for plaque control and managing gingivitis (Van der Weijden et al., 2015) or for treatment of periodontitis (Teughels et al., 2009). In addition, CHX is included in a wide range of oral care consumer products (Sanz et al., 2013; Cieplik et al., 2018b).

Therefore, the objectives of this work are to review the evidence for resistance in oral bacteria toward CHX and to summarize our understanding of potential resistance mechanisms toward CHX. This review further aims to raise awareness among the dental community about the potential risk of resistance toward CHX as well as concomitant cross-resistances toward antibiotics and to propose future research directions related to CHX resistance in oral bacteria.

## Chemistry and Historical Aspects

**FIGURE 1:**
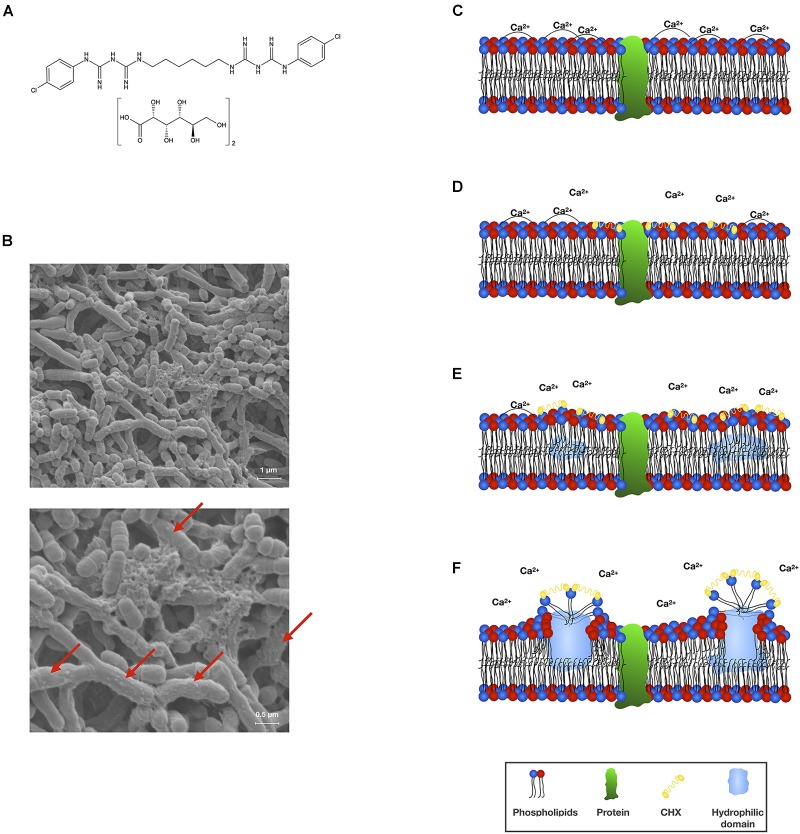
**(A)** Chemical structural formula of CHX digluconate. **(B)** Scanning electron microscopic (SEM) visualization of an *in vitro* polymicrobial biofilm comprising *Actinomyces naeslundii*, *Actinomyces odontolyticus*, and *S. mutans* (methodology as described in Cieplik et al., 2018b,c) following treatment with CHX (0.2%; 10 min). Vesicle-like structures on the surfaces of bacterial cells indicate membrane damage (indicated by red arrows). SEM images are reprinted from Cieplik et al. (2018b). **(C–F)** Scheme depicting the mode of action of CHX toward bacterial cytoplasmic membranes. The bacterial cytoplasmic membrane carries a net negative charge and is composed of a phospholipid bilayer with embedded proteins. The phospholipid bilayer is stabilized by divalent cations such as Ca^2+^ and forms a hydrophobic environment, which is essential to moderate the functionality of the embedded proteins **(C)**. CHX (as a cationic agent) binds to the negatively charged bacterial cell surface and initially interacts with the cytoplasmic membrane. Thereby, CHX bridges between pairs of phospholipid headgroups and displaces the associated divalent cations **(D)**. Progressive decrease in fluidity of the outer phospholipid layer with creation of hydrophilic domains within the bilayer affecting the osmoregulation and metabolic activity of the cytoplasmic membrane and its associated enzymes **(E,F)**. This scheme was adopted and modified from Gilbert and Moore (2005).

Chlorhexidine is a symmetric bis-biguanide molecule comprising two chloroguanide chains that are connected by a central hexamethylene chain and carries two positive charges at physiological pH (see Figure 1A for structural formula). It acts as a strong base and is practically insoluble in water but reacts with acids to form salts that exhibit varying water solubility characteristics (Davies et al., 1954; Denton, 2001; Lim and Kam, 2008). Today, mostly the digluconate or diacetate salts are used, whereby the water solubility of CHX digluconate is considerably higher than that of CHX diacetate (50% w/v vs. 2% w/v) (Kampf, 2018b). For the sake of clarity, in the present paper the acronym CHX will be used for all of its salts.

Chlorhexidine was first synthesized in Great Britain in the early 1950s within the scope of a broad screening exercise for active agents against malaria by Imperial Chemical Industries (Manchester, United Kingdom) (Lim and Kam, 2008; Kampf, 2016). It was described by Davies et al. (1954) under the name “Hibitane^®^” as “a new antibacterial agent of high potency” using its diacetate and dihydrochloride salts to ensure water solubility. Since then CHX has extensively been used in various medical fields (e.g., urology, gynecology, ophthalmology, otolaryngology) as well as for disinfection of surgical environments before it was introduced to dentistry in the late 1960s (Gjermo, 1974).

## Antibacterial Mechanism of Action

In general, it is often difficult to distinguish the primary mechanism of action of a given antiseptic or biocide from secondary effects that are merely a consequence of the action (Maris, 1995). CHX is usually described to act as a bacteriostatic agent at low concentrations and to be bactericidal at higher concentrations (Jones, 1997). Its antibacterial mechanism of action is described as damage of bacterial membranes and subsequent leakage of cytoplasmic components (see Figure 1B for scanning electron microscopic images of CHX-mediated damage to bacterial cell surfaces) (McDonnell and Russell, 1999; Gilbert and Moore, 2005). First-line targets of CHX (at lower concentrations) are cytoplasmic membrane integrity as well as function of membrane-bound enzymes (see Figure 1C–F for a scheme describing the detailed interaction of CHX with bacterial cytoplasmic membranes), while secondary effects (at higher concentrations) are cytoplasmic leakage and, ultimately, coagulation and precipitation of intracellular constituents such as proteins and nucleic acids (Denyer and Stewart, 1998; McDonnell and Russell, 1999; Gilbert and Moore, 2005). As cytoplasmic membranes are the main action sites of CHX, the outer membrane in Gram-negative bacteria may act as a permeability barrier for CHX and limit its antibacterial efficacy. For instance, cationic CHX molecules may be “locked up” in the outer membrane due to interactions with negatively charged lipopolysaccharide-moieties and thus may not be able to even reach the cytoplasmic membrane (Cheung et al., 2012). Furthermore, outer membrane vesicles released by Gram-negative bacteria like *Porphyromonas gingivalis* may bind CHX, thus allowing protection of bacteria (Grenier et al., 1995). As there is not much data on the actual role or influence of the outer membrane in Gram-negative bacteria with regard to the action of CHX, this point merits closer attention.

Studies of the effects of CHX at sub-inhibitory, bacteriostatic, concentrations are very scarce. Analogous to QACs or antibiotics at sub-inhibitory concentrations, the effects of CHX at such concentrations may be far more complicated including multiple processes such as loss of osmoregulation around the cytoplasmic membrane, disturbance of processes related to transport and respiratory activity, dissipation of proton motive force and oxidative stress, which triggers SOS responses and in turn induces error-prone DNA replication leading to mutations and gene transfers (Andersson and Hughes, 2014; Tezel and Pavlostathis, 2015).

## Resistance Mechanisms

Intrinsic resistance is defined as natural property of an organism, while acquired resistance is resulting from genetic changes and arising either by mutation or by the acquisition of the genetic material, e.g., *via* plasmids (Russell, 1995). Furthermore, one has to clearly distinguish between phenotypic adaptation, which is reversible when the exposure to the agent ends, and acquired resistance, which is genetically defined and therefore stable (Meyer and Cookson, 2010). Resistance must also be differentiated from tolerance, whereby resistance means the inherited ability of microorganisms to survive high concentrations of an antimicrobial drug (quantified by the minimum inhibitory concentration, MIC), while tolerance describes the ability to withstand a transient exposure to high concentrations of an antimicrobial (which otherwise would be lethal) without a change in MIC mostly due to the deceleration of metabolic processes (Brauner et al., 2016). In contrast, persistence describes the presence of a sub-population that is able to survive a treatment with concentrations higher than the MIC of a given antimicrobial despite the population being clonal (Brauner et al., 2016). These terms have often been used synonymously in the literature (especially in older studies), which hampers differentiation.

Furthermore, the definition of “resistance” toward antiseptics is not entirely clear (Chapman, 2003; Sheldon, 2005; Maillard, 2007). There are internationally recognized and standardized methods for susceptibility testing of antibiotics with defined breakpoints by means of which given isolates can be classified as resistant, intermediate or susceptible. This practically means that given isolates whose MIC values exceed the breakpoint concentrations are deemed resistant to a given antibiotic (Chapman, 2003). In contrast, similar frameworks are missing for susceptibility testing of biocides like CHX and there is a lack of well-defined MIC cut-off values indicating resistance (Vijayakumar and Sandle, 2018). While *in vitro* methods originally developed for systemic antibiotics (e.g., MICs) are still used for antiseptics, their results must be interpreted with caution (Sheldon, 2005). In this context, measurable MIC increases by a given factor (often 4- to 16-fold) have been considered relevant and may be seen as a parallel definition of “resistance” (Chapman, 2003), while on the other hand “in-use” concentrations of these biocides may be much higher than the measured MICs (Maillard, 2007; Vijayakumar and Sandle, 2018). Despite these limitations, investigation of MICs can still be valuable for antiseptics for studying potential resistance mechanisms *in vitro* (Maillard, 2007).

Intrinsic resistance to CHX is known from bacterial spores and mycobacteria and is due to their outer cell layers which form an impermeable barrier to the ingress of CHX molecules (Horner et al., 2012). Acquired genetically defined mechanisms conferring resistance toward CHX include multidrug efflux pumps and cell membrane changes (Jaglic and Cervinkova, 2012; Kampf, 2018b). The latter have been suspected to be responsible for CHX resistance in *Pseudomonas stutzeri*, where changes in the outer membrane protein and lipopolysaccharide profiles were found in CHX-resistant strains as compared to CHX-sensitive ones (Tattawasart et al., 2000b). Accordingly, electron-microscopic investigations confirmed that treatment with CHX led to considerably greater morphological changes in CHX-sensitive cells, while CHX-resistant cells showed no structural damage. Furthermore, it was confirmed by energy-dispersive spectroscopy that there was less uptake of CHX in resistant cells (Tattawasart et al., 2000a). The CHX-resistant strains also showed decreased susceptibility toward different classes of antibiotics which was suggested to be due to a non-specific decrease in cell permeability limiting uptake of chemically unrelated molecules into the resistant cells (Tattawasart et al., 1999).

**FIGURE 2:**
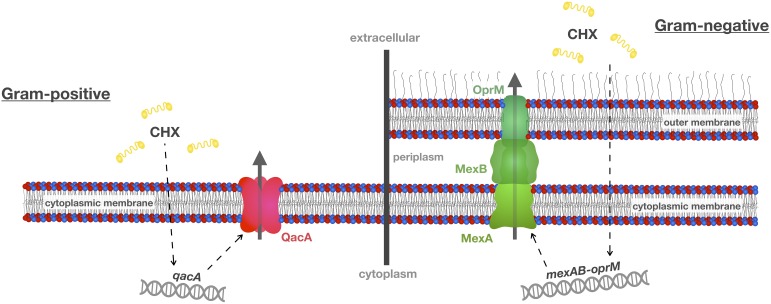
Efflux mechanisms conferring resistance toward CHX. A common resistance mechanism toward antibacterial agents such as CHX is up-regulation of multidrug efflux pumps. This scheme shows two well-known efflux systems, i.e., qacA in Gram-positive *S. aureus* and mexAB-oprM in Gram-negative *P. aeruginosa*. These efflux pumps may also be present in oral bacteria and recognize not only CHX as their substrate but also other antiseptics and antibiotics, and, thus, may contribute to cross-resistances between CHX and antibiotics. This scheme was adopted and modified from Venter et al. (2017).

Multidrug efflux pumps are membrane proteins that contain multiple transmembrane domains forming channels to remove toxic substances from the cytoplasm and the cytoplasmic membrane (see Figure 2 for schematic depiction of two exemplary efflux systems conferring CHX resistance in Gram-positive or Gram-negative bacteria, respectively) (Wassenaar et al., 2015). For instance, the plasmid-borne *qac* (standing for “quaternary ammonium compound”) gene family (e.g., *qacA*, *qacB*, *qacC*; *qacA*, and *qacB* usually recorded as *qacA/B* and *qacC* synonymously termed *smr*) is well known in Gram-positive bacteria (mainly in staphylococci). These *qac* genes encode for Qac efflux proteins that belong to the “Major Facilitator Superfamily” (MFS, i.e., QacA/B) or to the “Small Multidrug Resistance” (SMR) family (i.e., Smr) and have cationic biocides like CHX as substrates (Littlejohn et al., 1991; Poole, 2007; Buffet-Bataillon et al., 2012; Jaglic and Cervinkova, 2012; Liu et al., 2015; Kampf, 2016). Accordingly, introduction of a CHX-based surface antiseptic protocol in an intensive care unit with methicillin-resistant *Staphylococcus aureus* (MRSA) prevalence of about 20% was found to lead to an immediate and sustained reduction in transmission of susceptible MRSA strains while strains carrying *qacA/B* genes were not affected after introduction of this protocol (Batra et al., 2010). In contrast, a recent study found no correlation in *S. aureus* isolates between susceptibility toward CHX and carriage of *qacA/B* genes (Hardy et al., 2018). Interestingly, when comparing two pairs of isolates with fourfold differences in susceptibility to CHX (as shown by minimum bactericidal concentrations, MBCs) but very close relation according to phylogeny data, single-nucleotide polymorphisms within chromosomal efflux systems encoded by *norA* or *norB* were identified indicating that *norA*/*norB* may functionally complement *qacA*/*qacB* in these strains (Hardy et al., 2018).

In Gram-negative bacteria, efflux proteins from the SMR family (e.g., QacE, QacEΔ1, QacF, QacG) and from the “Resistance-Nodulation-Division” (RND) superfamily (like the Mex efflux systems in *P. aeruginosa*) have been described (Poole, 2007; Liu et al., 2015). For instance, the activity of the MexCD-OprJ multidrug efflux system was found to be upregulated in *P. aeruginosa* upon exposure to sub-inhibitory concentrations of CHX or BAC (Morita et al., 2014). Expression of this efflux gene was found in various mutants, suggesting that MexCD-OprJ is a determinant of CHX resistance (Fraud et al., 2008). Furthermore, the four RND superfamily efflux pumps MexAB-OprM, MexCD-OprJ, MexEF-OprN, and MexXY-OprM are also well recognized to confer resistance to fluoroquinolones (Morita et al., 2014). Likewise, the “Proteobacterial Antimicrobial Compound Efflux” (PACE) family is wide-spread in Gram-negative bacteria conferring resistance to a wide range of biocides (Hassan et al., 2018) whose first member, the active CHX efflux protein AceI, was identified by investigating the transcriptomic response in *Acinetobacter baumannii* toward CHX (Hassan et al., 2013). In the same study, it was also found that expression of the RND efflux protein AdeAB was even more up-regulated upon CHX exposure (Hassan et al., 2013).

The close association frequently found between antibiotic and antiseptic resistance may be explained by the fact that genetic determinants of resistance are commonly linked to each other; accordingly, the *qac* genes are frequently located on plasmids with various other resistance genes (Jaglic and Cervinkova, 2012). For example, a clinical strain of *S. aureus* with high-level resistance to vancomycin isolated in June 2002 harbored a 57.9 kb multi-resistance conjugative plasmid, pLW1043, comprising elements that encode for resistance toward vancomycin (*vanA*), trimethoprim (*dfrA*), β-lactams (*blaZ*), aminoglycosides (*aacA-aphD*) as well as antiseptics (*qacC*) (Weigel et al., 2003). Likewise, the plasmid pSAJ1 from a methicillin- and gentamicin-resistant strain of *S. aureus* isolated in the 1980s conferred resistance toward CHX (most likely due to *qacA*) along with resistance toward kanamycin, gentamicin, tobramycin, amikacin, BAC, acriflavine, and ethidium bromide (Yamamoto et al., 1988).

Recently, it was found that adaptation of clinical *Klebsiella pneumoniae* isolates to CHX *in vitro* can lead not only to stable resistance toward CHX but also cross-resistance toward the last line antibiotic colistin (Wand et al., 2017). Whole genome sequencing revealed mutations in *phoPQ* and/or *smvR*, whereby the *smvR* mutation in turn may lead to upregulation of *smvA* which encodes for expression of SmvA, an efflux pump belonging to the MFS, which was suggested to play a major role in CHX resistance of *K. pneumoniae* strains. The cross-resistance toward colistin upon adaptation to CHX was likely due to the mutation in PhoPQ affecting the regulatory targets *pmrD* and *pmrK*. The operon *pmrK* functions to alter lipopolysaccharides by replacement of phosphate groups by 4-amino-4-deoxy-L-arabinose, which in turn reduces the net negative charge of lipid A and results in a reduction of its binding affinity to colistin (Wand et al., 2017).

## Evidence for Resistance Toward CHX in Oral Bacteria?

Chlorhexidine has been extensively used in dental practice since 1970, when Löe and Schiøtt (1970) described total inhibition of plaque formation and gingivitis development in patients applying a 0.2% CHX mouth rinse twice daily despite stopping all other oral hygiene measures (e.g., tooth brushing) (see Gjermo, 1974). Already in 1972 two studies were published reporting clinical isolates of *Streptococcus sanguinis* that showed slightly reduced susceptibility toward CHX after long-term use of CHX-containing mouth rinses, which was, however, considered to be “relatively inconspicuous” (Emilson et al., 1972; Schiøtt and Löe, 1972). Isolates of *S. sanguinis* with reduced susceptibility to CHX were also found in a later clinical study upon daily tooth brushing with 0.5% CHX-containing gel, whereby the authors concluded that it was unclear whether this finding was a result of an adaptation toward CHX or due to a selection for less sensitive mutants within the original oral microbiota (Emilson and Fornell, 1976).

Westergren and Emilson (1980) described adaptation to CHX in three CHX-sensitive strains of *S. sanguinis* when grown *in vitro* as continuous cultures in a fermenter containing medium with increasing CHX concentrations. This resistance persisted after continuous growth in CHX-free medium and, interestingly, extracted DNA from the resistant mutants transformed competent sensitive *S. sanguinis* strains to increased CHX resistance. Therefore, they concluded that appearance of less susceptible isolates in the oral cavity after long-term use of CHX may be explained by genetic changes and not solely by selection for naturally occurring less susceptible strains (Westergren and Emilson, 1980). Likewise, when screening 315 isolates from subgingival plaque for their susceptibility to a mouth rinse containing 0.2% CHX, evidence for what the authors called “relative resistance” in different strains of *Streptococcus mitis*, *S. sanguinis*, and *Capnocytophaga* spp., was found (Wade and Addy, 1989). Maynard et al. (1993) reported significantly higher MICs for CHX in bacteria recovered from patients that had been using a 1% CHX toothpaste for 6 months as compared to those using a toothpaste without CHX. However, this finding was not thought to be of clinical significance (Maynard et al., 1993).

Passaging 24 periodontitis-associated bacteria (i.e., 10 oral streptococci, 8 *P. gingivalis* strains, 4 *Aggregatibacter actinomycetemcomitans* strains, and 2 enterobacteria) on agar plates containing sub-inhibitory concentrations of CHX resulted in a transitory moderate increase in the tolerance to CHX in five of the tested isolates (i.e., 1 oral streptococcus strain, 3 *P. gingivalis* strains, and 1 *A. actinomycetemcomitans* strain) after 25 passages, which vanished again after 50 passages in spite of the agar still containing sub-inhibitory concentrations of CHX (Eick et al., 2011). Kulik et al. (2015) reported two- to fourfold increased MICs in two out of five *P. gingivalis* strains after culturing for 20–30 passages in sub-inhibitory concentrations of CHX, whereas no increase in MIC was observed for two *Streptococcus mutans* and two *Streptococcus sobrinus* strains.

Recently, Kitagawa et al. (2016) found that repeated exposure of *Enterococcus faecalis* to CHX by serial passaging (10 cycles of exposure to CHX for MIC testing followed by re-growth in CHX-free medium) resulted in continuous increase in its MIC. The adapted cells showed increased surface hydrophobicity. Furthermore, SDS-PAGE of the adapted mutant strain revealed a novel protein of about 19-kDa (Kitagawa et al., 2016) that had also been found in vancomycin-resistant enterococci (Cho et al., 2008). The authors searched the proteome of *E. faecalis* 62 in the NCBI database for a candidate protein and reported that the most relevant protein in the 19-kDa range with regard to resistance was a 19.93-kDa MFS efflux pump protein (Kitagawa et al., 2016). This protein is unusually small for an MFS transporter and it is not clear whether it directly exports vancomycin. However, it is noteworthy that the candidate protein is a partial match to a larger 45-kDa MFS protein of *E. faecalis* V583, encoded by *ef2068* (Yan et al., 2015; Kitagawa et al., 2016).

Wang et al. (2017a) evaluated changes in MIC in eight common oral bacterial species over 10 passages of CHX-challenge and re-growth in CHX-free medium and reported adaptation in *Streptococcus gordonii*, *E. faecalis*, *Fusobacterium nucleatum*, and *P. gingivalis*. Further analysis of the adapted *S. gordonii* strain showed a delayed growth with prolonged log-phase and decelerated growth rate as compared to its parental strain indicating reduced metabolic activity that may be responsible for the reduced susceptibility toward CHX (Wang et al., 2017a).

In another recent study, bacteria were isolated from dental plaque from five healthy individuals to screen for strains resistant toward CHX (Saleem et al., 2016). CHX-resistant isolates also displayed variable resistance to a range of antibiotics including ampicillin, kanamycin, gentamicin and tetracycline. Exposure of the most resistant isolate, a strain identified as *Chryseobacterium indologenes*, to CHX (16 μg/mL) resulted in a 19-fold up-regulation of the gene CIN01S_RS05745 encoding expression of the HlyD-like periplasmic adaptor protein of a tripartite efflux pump. However, it was not investigated whether the other components of this efflux pump were upregulated as well (Saleem et al., 2016). The authors highlighted the requirement for increased vigilance for the presence of multidrug resistant bacteria within dental plaque and raised awareness of the potential risk of long-term use of oral care products containing antimicrobial agents such as CHX for the control of oral biofilms.

Interestingly, treatment with CHX has also been shown to induce the formation of antifungal-tolerant persister cells in *Candida albicans* biofilms *in vitro* (LaFleur et al., 2006). In another study, 150 isolates of *C. albicans* and *Candida glabrata* obtained from cancer patients, who were at high risk for the development of oral candidiasis and who had been treated with topical CHX once a day, were investigated in terms of the development of persisters (LaFleur et al., 2010). The authors found that persister cells are clinically relevant and that antimicrobial therapy with CHX and long-term carriage of *Candida* select for high-persister strains *in vivo*. There are also reports of the induction of *S. mutans* persisters by CHX in single-species biofilms (Wang et al., 2017b).

**FIGURE 3:**
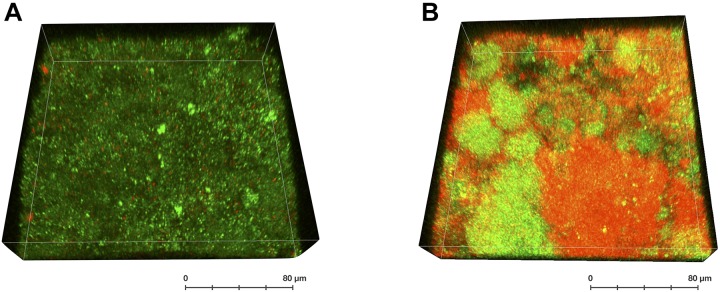
3D-reconstruction of a LIVE/DEAD-stained confocal image stack of a supragingival oral biofilm after treatment with CHX. The biofilm was formed *in situ* on a bovine enamel slab for 72 h (methodology as described in Al-Ahmad et al., 2015) and was either left untreated **(A)** or was treated with 0.2% CHX for 5 min **(B)**. Bacteria with intact (green; considered “live”) or compromised bacterial membranes (red; considered “dead”) are depicted indicating “pockets of viable cells” within the biofilm.

The increased clinical use of CHX makes it important to be alert to the possibility of the emergence of new clones with reduced susceptibility (Horner et al., 2012). It seems reasonable that microorganisms will be exposed to sub-inhibitory concentrations of antiseptics in a clinical environment (Block and Furman, 2002). Such sub-inhibitory concentrations can be reached in the oral cavity upon treatment with CHX, as the antimicrobial effect of CHX seems to be diminished in the presence of organic substance such as saliva or serum due to inactivation by salivary or serum proteins (Portenier et al., 2006; Abouassi et al., 2014). Also inside oral biofilms, treatment with CHX results in a concentration gradient from the biofilm surface toward its lower strata which may lead to biofilm layers with sub-inhibitory concentrations of CHX (Thurnheer et al., 2003). Accordingly, the limited antimicrobial effects of CHX in the inner layers of oral biofilms have been shown using confocal laser scanning microscopy in combination with LIVE/DEAD staining (see Figure 3) (Zaura-Arite et al., 2001; Karygianni et al., 2014; Al-Ahmad et al., 2016). Furthermore, starvation may play a major role in these biofilms, which may increase adaptation of the bacteria within the biofilm. It was shown that *S. mutans* exhibited significantly increased MBCs toward CHX under starvation conditions *in vitro* than in nutrient-rich medium (Tong et al., 2011).

## Conclusion and Future Directions

The question, whether there already is reason for concern with regard to enhanced tolerance or even resistance toward CHX in oral bacteria, cannot be answered that easily and must be treated in a more differentiated way: On the one hand, CHX is still very effective in its various fields of clinical application, e.g., intensive care, hand soaps and as an oral antiseptic. On the other hand, there unquestionably are reports about emergence of isolates exhibiting enhanced tolerance toward CHX. True resistance toward CHX will have horrendous consequences for infection control. For example, Copin et al. (2019) recently reported the spread of a unique USA300 clone of community-acquired MRSA in an Orthodox Jewish Community in Brooklyn. Genetic analyses showed that a metabolic change (mutation in *pyrA*) and acquisition of a clone-specific prophage (Φ11) probably have primed the clonal variant for success by promoting colonization and abscess formation. However, it was also found that emergence of a dominant clone coincided with acquisition and evolution of a plasmid (pBSRC1) with genes conferring resistance to CHX (*qacA/B*) and mupirocin (*mupA*), strongly suggesting that resistance toward antimicrobials used for decolonization therapy like CHX and mupirocin were key elements in the spread of this clone (Copin et al., 2019). Moreover, low level exposure to CHX (as potentially occurring in deeper layers of oral biofilms) may result in development of cross-resistances toward antibiotics as it has already been described in biocide-sensitive strains from organic foods (Kampf, 2018a). As there currently is little awareness about the potential risks accompanying the widespread use of CHX in dentistry, this research subject should be particularly highlighted as a hot topic in dental research. From the authors’ point of view, there are two key questions relating to CHX resistance that urgently need to be addressed:

• Does the widespread use of CHX lead to an enrichment of resistant strains in oral biofilms and does it further encourage the development of cross-resistances in oral biofilms?
• What are the molecular mechanisms conferring CHX resistance in oral bacteria?

The emergence of resistant bacteria as a result of the widespread use of CHX in dental practice has not been studied systematically so far. Culturing biofilms in oral *in situ* biofilm models and treating them with CHX sequentially would give insights into ecological changes of the microbiota of the oral biofilm after long-term treatment with this antimicrobial agent. In this light, it will be important to analyze the impact of CHX by microbiome analyses by high-throughput sequencing methods as well as by culture techniques that can distinguish between viable and non-viable cells. In addition, subsequent testing of antibiotic resistance in representative oral isolates may facilitate the study of potential effects of CHX on cross-resistance toward antibiotics and emergence of multidrug resistant bacteria. What remains unclear is the clinical relevance of persister cells in oral biofilms which have frequently been treated with CHX. The research on persister development in oral bacteria under the pressure of CHX treatment is still in its early stage.

**FIGURE 4:**
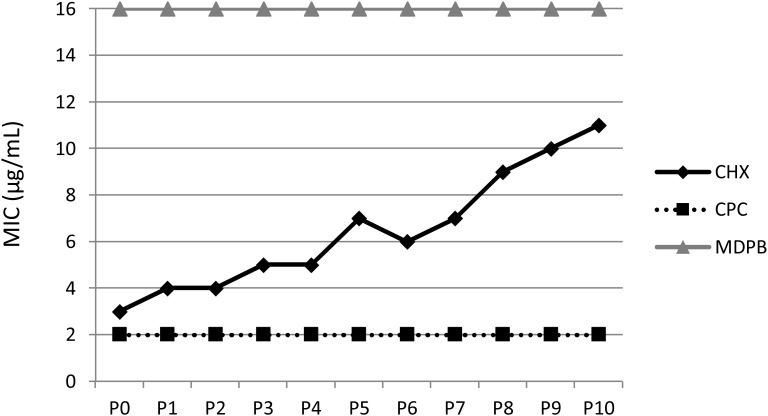
Multiple passaging of bacteria under CHX-treatment *in vitro*. MICs of CHX, cetylpyridinium chloride (CPC) and methacryloyloxydodecylpyridinium bromide (MDPB) repeatedly performed from passages 0–10 (P0–P10) against *E. faecalis*. This figure is reprinted from Kitagawa et al. (2016) with kind permission from the publisher.

Furthermore, novel techniques like transposon sequencing (Tn-seq) are now available that can be used to identify key genetic mechanisms of CHX resistance in oral bacteria by determining gene disruptions differentially represented in mutant populations on a genome-wide scale (van Opijnen et al., 2009; Burby et al., 2017). For this purpose, resistant strains must either be isolated from patient samples or induced *in vitro* by multiple passaging of bacteria under CHX-treatment (see Figure 4), whereby the latter has the advantage that the mutant strain exhibiting phenotypic adaptation to CHX can be compared to the wildtype strain. Finally, it is important to consider the respective fitness cost of a given mutation related to CHX resistance as the magnitude of this cost is the main biological parameter determining the rate of development of resistance (Andersson and Hughes, 2010; Cai et al., 2017).

## Data Availability

All datasets generated for this study are included in the manuscript.

## Author Contributions

FC and AA-A took the lead in writing the manuscript. NJ, WB, TM, and EH provided the critical feedback and reviewed the manuscript for high intellectual content.

## Conflict of Interest Statement

The authors declare that the research was conducted in the absence of any commercial or financial relationships that could be construed as a potential conflict of interest.
